# Consumption of anthocyanin-rich beverages affects Nrf2 and Nrf2-dependent gene transcription in peripheral lymphocytes and DNA integrity of healthy volunteers

**DOI:** 10.1186/s13065-020-00690-6

**Published:** 2020-05-29

**Authors:** Isabel Anna Maria Groh, Tamara Bakuradze, Gudrun Pahlke, Elke Richling, Doris Marko

**Affiliations:** 1 Faculty of Chemistry, Department of Food Chemistry and Toxicology, University of Vienna, Währingerstraße 38, 1090 Vienna, Austria; 2 Present Address: Department of Experimental and Clinical Pharmacology and Pharmacogenomic, University of Tuebingen, Wilhelmstraße 56, 72072 Tuebingen, Germany; 3 Department of Chemistry, Division of Food Chemistry and Toxicology, Technische Universitaet Kaiserslautern, Erwin-Schrödinger-Straße 52, 67663 Kaiserslautern, Germany

**Keywords:** Anthocyanins, Nrf2, DNA integrity, Human intervention study, Peripheral blood lymphocytes

## Abstract

Recently, we demonstrated that the consumption of a bolus of bilberry extract modulates the transcription of Nrf2-regulated genes in peripheral blood lymphocytes (PBL) of healthy volunteers, accompanied by decreased DNA-damage. In the present study, we addressed the question whether consumption of consumer-relevant amounts of anthocyanin-rich beverages can achieve similar effects. The impact of three different anthocyanin-rich beverages on Nrf2-dependent gene transcription as well as and the status of DNA-damage in whole blood was investigated. After a polyphenol-reduced diet, five healthy male subjects consumed a bolus (700 mL) of respective test beverages with blood sampling up to 8 h after intake. All beverages affected the transcription of Nrf2, HO-1 and NQO-1, but showed different potencies and persistence of effects. Consumption of red fruit juice significantly reduced total DNA strand breaks (with formamidopyrimidine-DNA-glycosylase-(fpg) treatment) after 8 h in blood samples of the volunteers, suggesting antioxidant and DNA protective effects, albeit transcript levels of Nrf2-dependent genes had reached the basal state. The amount of basic DNA strand breaks (damage without oxidative DNA strand breaks) remained unchanged during the monitoring period. In contrast, a beverage prepared from grape skin extract significantly increased basic and total DNA strand breaks 2 h after intake, underlining the necessity of further investigations regarding composition, safety and consumer´s acceptance of respective products to exclude undesired adverse effects. Consumption of a bolus of anthocyanin-rich beverages affected Nrf2 and Nrf2-dependent gene transcription in human PBL and DNA integrity, which is indicative for systemic effects.

## Introduction

A misbalance in the homeostasis of reactive oxygen species (ROS) is known to contribute to the pathogenesis of a spectrum of life-style and nutrition-associated chronic diseases [1]. ROS can damage all cellular macromolecules, including proteins, lipids, and DNA. Foods rich in antioxidants are able to scavenge ROS or to suppress their formation. In addition, multiple biological activities such as modulation of cellular defense or maintenance of DNA integrity are well known properties of antioxidants [2]. Especially, regarding anti-oxidative effectiveness, the class of anthocyanins is intensively studied. Anthocyanins represent a class of colored plant constituents, which occur in many fruits and vegetables of the daily diet like grapes, berries and respective products such as juices or red wine [3–5]. In several in vitro studies and even in human intervention studies, anthocyanin-rich preparations have been shown to reduce oxidative stress markers [6–10]. In healthy volunteers, consuming a bolus of a bilberry extract, higher levels of metabolites than parent anthocyanins in plasma and urine were measured, emphasizing the important role of the colon in this context [11]. Antioxidant activity of food constituents is not exclusively limited to direct scavenging of ROS. A different approach is the induction of antioxidant defense by activating the redox-sensitive Nrf2-pathway, steering among others the expression of antioxidative enzymes such as NAD(P)H quinone oxidoreductase 1 (NQO-1) and heme oxygenase 1 (HO-1). The transcription factor Nrf2 is a key element in the intracellular anti-oxidant defense [12]. In its inactive form, Nrf2 is bound to the actin-coupled Kelch-like ECH-associated protein 1 (Keap1) in the cytosol of the cells. Through the impact of ROS, the interaction between Nrf2 and Keap1 is disrupted, thus enabling the translocation of Nrf2 into the nucleus. After binding to small-Maf proteins (sMaf), the resulting complex binds to antioxidant responsive elements (ARE), regulating the transcription of a battery of genes including important phase-II-enzymes as well as enzymes crucial for antioxidant defense like NQO-1 and HO-1 [2, 13–16]. In a human pilot intervention study, we demonstrated recently that the consumption of a bolus of bilberry extract modulates the transcription of Nrf2, NQO-1 and HO-1 in peripheral blood lymphocytes (PBL) of the volunteers, accompanied by a decrease of total DNA damage [6]. Likewise, a significant reduction of basic and total DNA strand breaks was reported in a human intervention study with healthy male volunteers after 4 weeks’ consumption of 700 mL anthocyanin-rich fruit juice [9]. However, in the latter study only the impact on DNA integrity in the PBL of the volunteers was monitored, leaving open the question if the observed effects are associated with an impact on the Nrf2-pathway.

In the present pilot intervention study, we addressed the question whether induction of Nrf2-dependent gene expression and a respective decrease of DNA damage are only possible with highly concentrated extracts or whether already a single bolus of consumer-relevant anthocyanin-rich juice is sufficient to achieve a significant modulation of the Nrf2-dependent protective system. We determined the effect of three different anthocyanin-rich beverages (bilberry drink produced from two different amounts of bilberry juice, fruit juice blended from red berries, and beverage enriched with grape skin extract) on the transcription of Nrf2, HO-1 and NQO-1 in PBL and on DNA integrity within 8 h after consumption.

## Results

### Characterization of study beverages

The composition of the beverages used in the study are presented in Table 1. Beverage 3 (bilberry juice) contained comparably high concentrations of total anthocyanins (2454 ± 29.1 mg/L) and total phenolics (4.1 g/L), but was diluted afterwards. In contrast, in beverage 1 (red fruit juice) 332 ± 48.8 mg/L and 3.0 g/L of anthocyanins and total phenolics were found, respectively. Beverage 2 enriched with grape skin extract showed the lowest concentration of total anthocyanins with 239.1 ± 5.1 mg/L.

**Table 1:** Characterization of beverages used in the pilot intervention trial

Parameters	Beverage 1 (red fruit juice)	Beverage 2 (with grape skin extract)	Beverage 3 (bilberry juice^a^)
TEAC	30 mmol/L	na	na
Total phenols (mg/L gallic acid equivalents)	3.0 g/L	na	4.1 g/L
Total anthocyanins (mg/L cyanidin-3-*O*-glucoside equivalents)	332.0 mg/L	239.1 mg/L	2454 mg/L
Citric acid	6.0 g/L	1.5 g/L	11.15 g/L
Vitamin C	401 mg/L	na	na
BRIX	13.0°	13.0°	9.75°
Fruit content	100%	20 g extract/L	100%

Beverage 1 (red fruit juice), 2 (juice with grape skin extract) and 3 (bilberry juice)

*na* not analyzed

^a^Bilberry juice (100%) was used to prepare the two beverages used in the human intervention study (see Table 2)

**Fig. 1:**
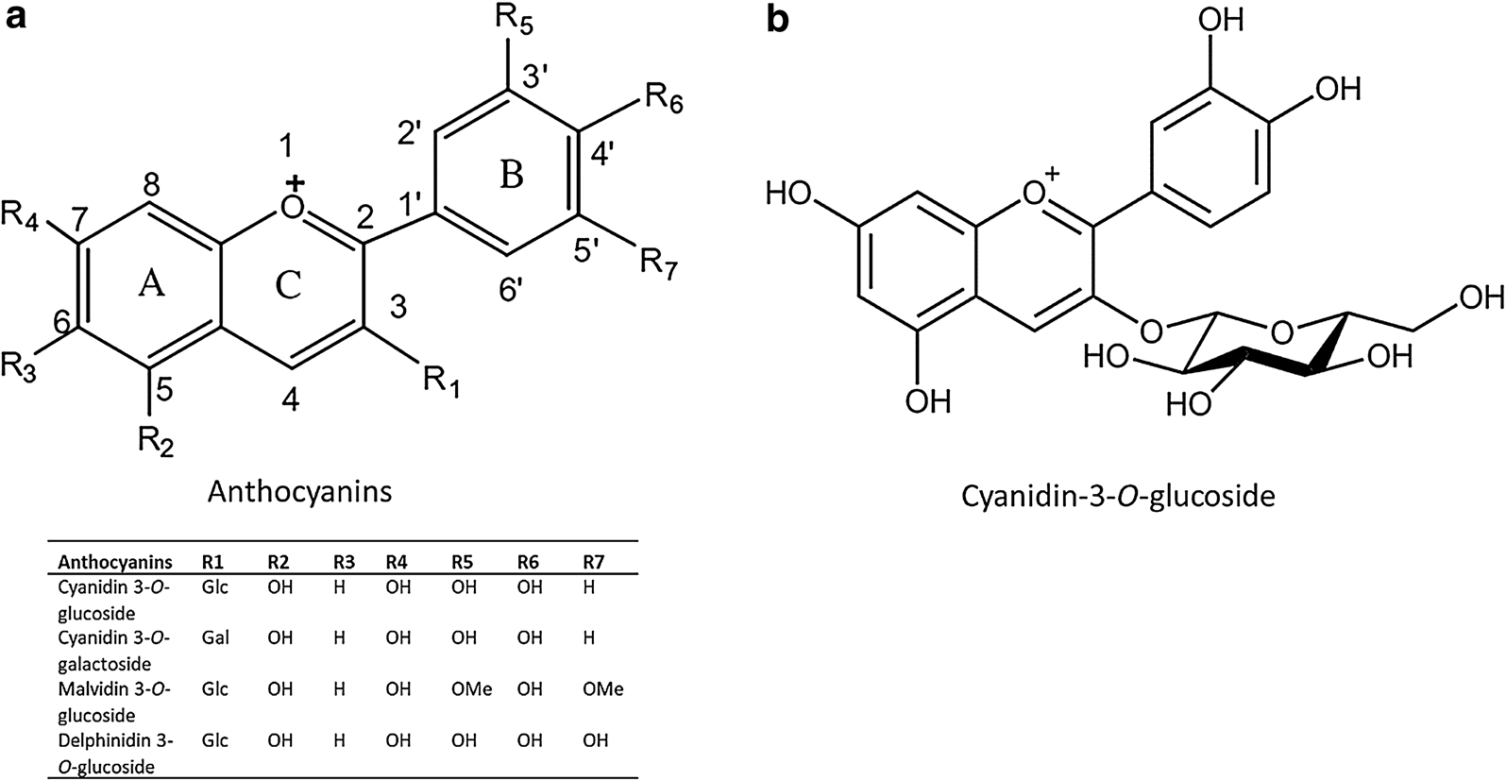
Chemical structure of anthocyanins [17] and substitution pattern of representative anthocyanins (**a**) and cyanidin-3-*O*-glucoside (**b**) as the measured anthocyanin equivalent in the test beverages

The amounts of anthocyanins consumed with the respective beverages (700 mL) and their °Brix values are summarized in Table 2. The structure of the anthocyanins and especially of cyanidin-3-*O*-glucoside (Cy-3-glc) as an anthocyanin-equivalent measured in the test beverages is shown in Fig. 1. The two bilberry drinks (beverage 3a and 3b) were standardized by the addition of sucrose so that all consumed beverages exhibited the same Brix values (13° Brix). Since the bilberry juice was tested in two different concentrations, the consumed total anthocyanin levels were 245.4 mg/700 mL for beverage 3a and 736.5 mg/700 mL for beverage 3b, respectively. The intake of red fruit juice and the beverage enriched with grape skin extract provided 232.4 mg/700 mL and 167.4 mg/700 mL anthocyanins, respectively.

**Table 2:** Anthocyanin content (mg/700 mL) in consumed study beverages and ° Brix values

Study beverages	Anthocyanin content (cyanidin-3-*O*-glucoside equivalents) (mg/700 mL)	° Brix
Beverage 1 (red fruit juice)	232	13
Beverage 2 (with grape skin extract)	167	13
Beverage 3a (bilberry drink^a^)	245	13
Beverage 3b (bilberry drink^b^)	736	13

Anthocyanin content (mg/700 mL) in consumed study beverages are calculated on the basis of total anthocyanin concentrations in mg/L cyanidin-3-*O*-glucoside equivalents

^a^100 mL bilberry juice with 600 mL water-sucrose solution

^b^300 mL bilberry juice with 400 mL water-sucrose solution

### Nrf2/ARE-dependent gene transcription

**Fig. 2:**
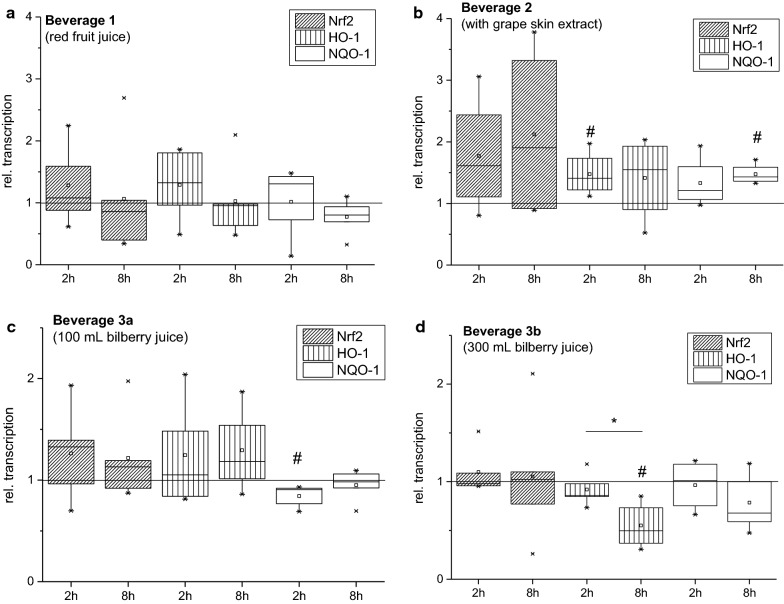
Modulation of the Nrf2, NQO-1 and HO-1 gene transcription in PBL of five healthy male participants of the human pilot intervention trial analyzed by qPCR; **a**: beverage 1 (red fruit juice); **b**: beverage 2 (with grape skin extract); **c**: beverage 3a (100 mL bilberry juice + 600 mL water-sucrose solution); **d**: beverage 3b (300 mL bilberry juice + 400 mL water-sucrose solution). The data, analyzed in triplicate, are presented as BOX-diagrams, values are mean ± SD; normalized to β-actin and GAPDH transcription and presented as relative transcription of baseline; significant differences from baseline were calculated using the Kruskal–Wallis ANOVA (^#^p < 0.05) and differences from time points were calculated using the Kruskal–Wallis ANOVA (*p < 0.05)

Potential modulatory effects on Nrf2-signaling by the consumption of three different anthocyanin-rich beverages in consumer-relevant amounts were investigated in isolated PBL from blood samples of 5 male volunteers. According to previous results of a short-term human pilot intervention trial in females, using a bolus of bilberry pomace extract [6], HO-1, NQO-1 and the central regulator Nrf2 itself were selected as potential marker genes. Transcript levels of the redox sensor Nrf2 itself were not significantly affected by the consumption of any applied beverage, neither after 2 h nor after 8 h of intake (Fig. 2). Also, transcript levels of HO-1 were not significantly impaired by the consumption of beverage 1 (red fruit juice) or 3 (both dosages of the bilberry drink) after 2 h of intake (Fig. 2a, c, d).

In contrast, beverage 2 comprising grape skin extract increased HO-1 mRNA with statistical significance, although to less than 1.5-fold increase and thus only to a minor extend (Fig. 2b). After 8 h of red fruit juice consumption (beverage 1) the HO-1 mRNA returned from a moderate yet statistically not significant increased level to its basal level (Fig. 2a), whereas the level appeared unaffected after intake of beverage 2. Consumption of beverage 3b (bilberry drink at the highest concentration) caused a significant reduction in HO-1 transcripts after 8 h (Fig. 2d). NQO-1 transcription was affected by bilberry and grape skin containing beverage as well. Whereas in PBL of participants consuming beverage 3a (bilberry drink at low concentration) NQO-1 mRNA levels were significantly reduced after 2 h (Fig. 2c), the transcription levels were significantly increased in participants drinking beverage with grape skin after 8 h (Fig. 2b). In general, it was apparent that transcription of Nrf2, NQO-1 and HO-1 was by trend increased by the consumption of beverage with grape skin extract after 2 h and 8 h. The mRNA level of HO-1 was statistically significant increased after 2 h of consumption, whereas the transcript level of NQO-1 was significantly enhanced after 8 h of consumption (Fig. 2b).

The intake of beverages 3 (bilberry drinks) resulted in a different pattern of transcriptional effect for the two different concentrations. Consumption of the bilberry beverage 3a slightly enhanced the transcription of Nrf2 and HO-1, although not reaching statistical significance (Fig. 2c). In contrast, 2 h after consumption a significant decrease of the NQO-1 transcripts was detected, which was reverted to control level after 8 h. Consumption of the 700 mL beverage 3b failed to enhance Nrf2 transcription at both time points. After a trend towards decrease (2 h), the transcript level of HO-1 was significantly suppressed 8 h after consumption (Fig. 2d). No significant effects were observed for the transcription of NQO-1 with a trend towards a decrease of transcript levels for the longer time point.

### DNA strand breaks

**Fig. 3:**
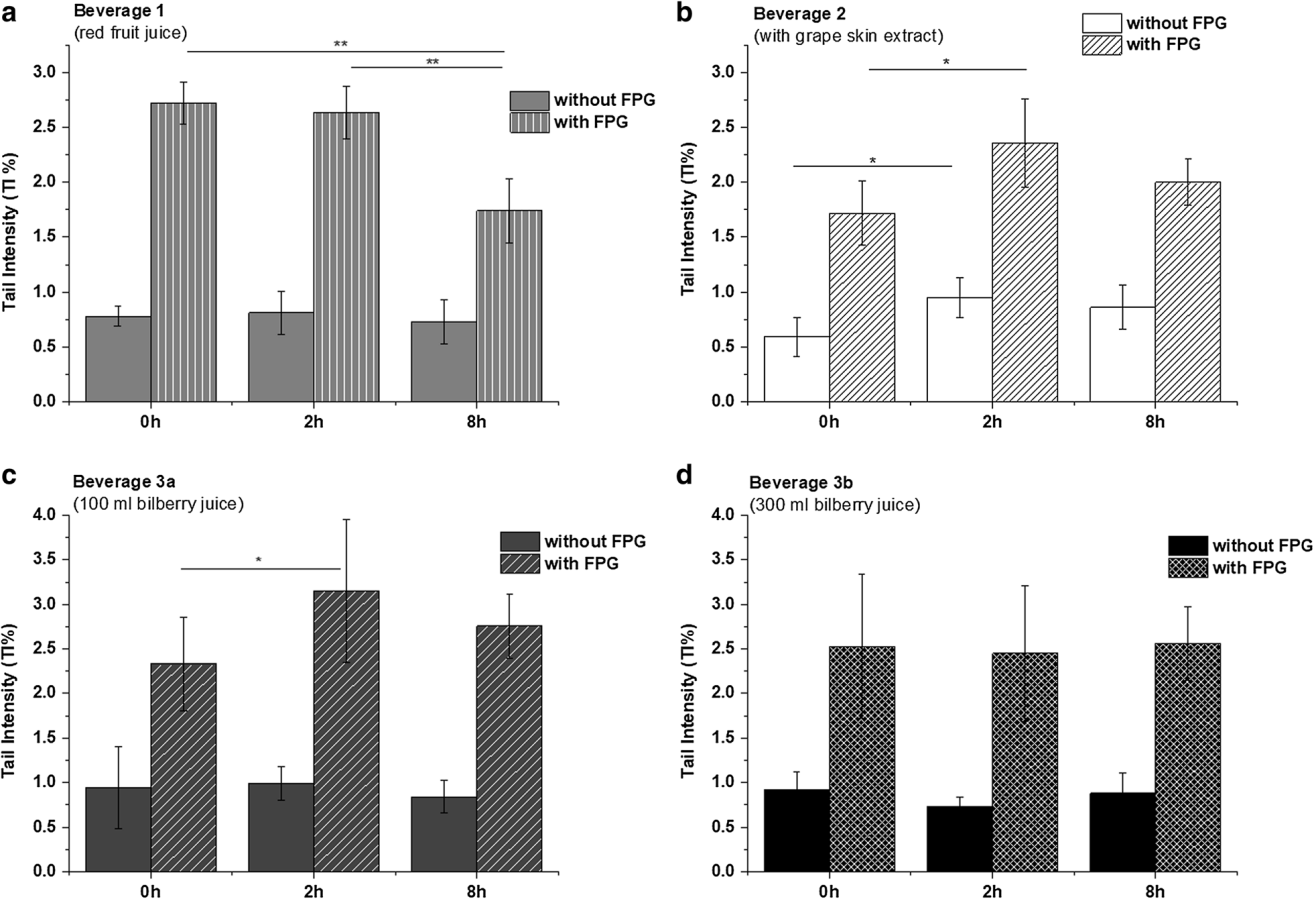
Modulation of DNA strand breaks in whole blood of the participants analyzed by comet assay after consumption of **a** beverage 1 (red fruit juice); **b** beverage 2 (with grape skin extract); **c** beverage 3a (100 mL bilberry juice + 600 mL water-sucrose solution); **d** beverage 3b (300 mL bilberry juice + 400 mL water-sucrose solution). The data are shown as TI (tail intensity) [%] Values are mean ± SD; significant differences are calculated using the Student’s *t* test and *Wilcoxon Signed Rank* Test **p < 0.01; *p < 0.05

The effects of the applied anthocyanin-rich beverages (beverage 1–3) on DNA integrity in whole blood samples was determined using the alkaline comet assay. The results showed different modulations of basic and total (basic strand breaks and oxidized bases) DNA strand breaks by all tested beverages (Fig. 3). The consumption of red fruit juice (beverage 1) demonstrated a significant reduction (p < 0.01) of total DNA damage 8 h after bolus juice ingestion, whereby the amounts of basic DNA strand breaks (without oxidative damage) were unchanged during the whole monitoring period of 8 h (Fig. 3a). A significant increase of basic and total DNA strand breaks was observed two hours after intake of beverage 2 prepared from grape skin extract (Fig. 3b) with a slight decrease 8 h after beverage intake (no significance level).

The bilberry drink in the low concentration (beverage 3a) significantly elevated the level of DNA strand breaks 2 h after consumption (Fig. 3c). In contrast, the bilberry drink with the higher anthocyanin concentration (beverage 3b) showed no clear modulation of DNA strand breaks (Fig. 3d). During the whole study period TI% of fpg-treated samples (total damage) were 4–5-fold higher, as compared to those of the respective basic strand breaks (without fpg). This indicates the presence of DNA oxidative damage (measured as fpg-sensitive sites).

In contrast to the protective effects of beverage 1 (red fruit juice), the intake of 700 mL beverage 2 comprising grape skin extract resulted in a transient increase of DNA strand breaks 2 h after consumption.

After ingestion of the bilberry beverage (beverage 3a and b) containing high amounts of anthocyanins (736 mg) no modulation of the relative level of DNA strand breaks was observable. In contrast, the consumption of lower amounts of anthocyanins in beverage 3a (245 mg) resulted even in a statistically significant increase of oxidative DNA damage (generation of fpg-sensitive sites) after 2 h, but returning to basal level after 8 h thus indicating only a transient effect.

## Discussion

The impact of consumption of three different anthocyanin-rich beverages (beverage 1–3) on the transcription of Nrf2 and selected Nrf2-dependent genes in PBL was investigated and compared with effects on DNA integrity, with special emphasis on the status of oxidative DNA damage. Important for the selection of the beverages was consumer acceptance and commercial availability and the anthocyanin content expressed as Cy-3-glc equivalents. One beverage had a low (239.1 mg/L, grape skin beverage), one 332.0 mg/L (red fruit beverage) and one a high (2454 mg/L, bilberry juice) anthocyanin concentration. The main goal of our pilot study was to identify the most potent anthocyanin-rich consumer relevant beverage with respect to protection against basal oxidative stress. In this context the role of the respective anthocyanin pattern, which indeed is quite different, was not in focus.

The oxidative marker genes (HO-1, NQO-1 and the central regulator Nrf2 itself) were selected according to previous results of a short-term human pilot intervention trial by Kropat et al. in females, using a bolus of bilberry pomace extract [6]. Normalized transcript levels revealed differences in potency and persistence of the modulatory effect. In the latter study, female healthy volunteers and volunteers with an ileostomy consumed a bolus of bilberry pomace extract. Already after 1 h of extract consumption, a significant impact on the Nrf2/ARE-mediated transcription was detected in healthy subjects. The transcript level of NQO-1 was moderately, but significantly, enhanced indicating the activation of the Nrf2/ARE pathway. Concomitantly, the mRNA level of HO-1 was decreased. The response pattern of the bilberry drinks (beverage 3a and 3b) reflect only partially the transcript pattern observed in Kropat et al. [6] for bilberry pomace extract. Indeed, in the present study a decrease in the transcription of HO-1 was achieved by the application of the bilberry beverage containing 300 mL of bilberry juice (beverage 3b) but not by consumption of the lower concentration (beverage 3a), arguing for a concentration-dependent effect. In contrast to the consumption of bilberry extract, both here applied bilberry beverages did not significantly enhance the transcription of NQO-1, but even transiently decreased the transcription level by application of the lower concentrated beverage (Fig. 2c). In comparison, the impact of the bilberry beverages on Nrf2/ARE-dependent gene transcription appear to be less pronounced compared to consumption of a bilberry pomace extract. Thereby not only the lower anthocyanin concentrations of the bilberry beverages (beverage 3a and b) but also potential matrix effects might have to be considered. Beyond doubt, the gender of the participants might also play a role for the differences in response. In our study we tested five healthy men according to the study of Weisel et al. [9]. In the literature it is already described that there are differences in the oxidative response of female and male after antioxidant consumption [18]. Furthermore, female gonadal hormones are discussed to be protective for oxidative stress and to have a smaller oxidative damage load than males for such as ischemic events [19].

The impact of transcriptional regulation of Nrf2/ARE-regulated genes is known to be cell type specific. In normal cells increased Nrf2 expression and activation of the Nrf2 pathway is assumed as a protective mechanism, moving the cell into a state of increased defense. Whereas overexpression of Nrf2 appears to play a role in resistance mechanisms to chemotherapeutics in cancer cells [20].

A counterbalanced level of HO-1 expression seems to be of substantial health impact. HO-1 catalyzes the rate-limiting step in the oxidative degradation of heme, but on the other hand this enzyme is also involved in the etiology of several diseases, such as tumor growth and Alzheimer’s disease [21]. NQO-1 catalyzes the reduction of potential redoxcyclers and is associated with chemoprotective properties [22]. The observed differential transcriptional response pattern of peripheral lymphocytes with respect to Nrf2/ARE-dependent genes depending on the applied beverages already indicated differences in the kinetics of the impact for the different genes (compare 2 h versus 8 h). These results raise the question on the impact of repeated (long-term) consumption.

Furthermore, the DNA integrity was measured after 2 h and 8 h of consumption of the different beverages, respectively. A single bolus consumption of beverage 1 (red fruit juice) was found to decrease the level of total DNA strand breaks in peripheral blood cells 8 h after consumption. These results appear to be in line with the results at the transcript level, tending towards enhanced gene transcript levels of Nrf2 and Nrf2-dependent genes 2 h after consumption (Fig. 2a). Beverage 2 containing grape skin extract significantly induced DNA strand breaks after 2 h of consumption (Fig. 3b). Thus, substantially enhanced Nrf2-mediated transcript levels might reflect the presence of potential stressors resulting in an activation of the Nrf2 pathway. These results together with indication of indisposition due to nausea underlines the necessity of further studies on the composition, safety and consumer´s acceptance of respective products using grape skin extract to exclude undesired adverse effects. Here it could be speculated, that stress might cause higher damages due to increase in respiration rates or due to other unknown reasons [23].

Bolus consumption of the beverage 3a (100 mL bilberry juice + 600 mL water-sucrose solution) failed to suppress DNA damage rather transiently enhanced the level of fpg-sensitive sites 2 h after consumption. Consumption of beverage 3b (300 mL bilberry juice + 400 mL water-sucrose solution) did not impact DNA integrity. The results for bilberry drinks are supported by the transcription analysis data of Nrf2 and Nrf2-dependent genes, showing no impact or rather increased transcript levels. Demonstrated for bilberry-based beverages, the results let assume that already differences in concentration of the same polyphenol mixture might result in a different activity profile.

The observed protective effects of beverage 1 (red fruit juice) on DNA integrity (Fig. 3a) was according to earlier findings by Weisel et al. [9]. In latter study, a significant reduction of DNA strand breaks was found in healthy volunteers (n = 18) after 4 weeks’ consumption of 700 mL red fruit juice with anthocyanin concentration of 197.9 mg/L (as cyanidin-3-*O*-glucoside equivalents). Likewise, Bub et al. reported a decrease of total DNA damage after 2 weeks’ consumption of anthocyanin-rich juice in healthy volunteers [10].

Moreover, the anthocyanin concentration in the applied bilberry drink was about 3.6 fold lower than in the bilberry pomace extract used by Kropat et al., who observed a significant reduction in DNA damage 2 h after consumption [6]. Thus, the ingested anthocyanin concentration might play an important role for the observed differences on DNA integrity of PBL. Nevertheless, other co-occurring polyphenols such as chlorogenic acids (called co-pigments), oligomeric or polymeric procyanidins, and especially the anthocyanin pattern in the different preparations should not be neglected, with higher complexity in bilberries. Recently, Juadjur et al. reported that in vitro a bilberry extract and the therefrom-isolated chlorogenic acid-rich fraction showed the most potent antioxidant activity in colon tumor cells whereas the polymeric and anthocyanin-rich fraction, in total, were less active [24]. The two bilberry drinks, which differed in anthocyanin concentrations (beverage 3a and b) already illustrate a different activity profile, so it is not remarkable that the consumption of an anthocyanin extract in a very high concentration showed different effects. Additionally, when consuming 10 g of bilberry pomace extract as reported by Kropat et al. [6], the total of ingested anthocyanins was 2.5 g, whereas in our present study the total anthocyanins consumed with the bolus were 232 mg for beverage 1 (red fruit juice), 167 mg for beverage 2 (with grape skin extract), and 245 and 735 mg with the beverages 3 (bilberry drinks), respectively.

## Conclusion

In conclusion, the consumption of a bolus of anthocyanin-rich beverages was sufficient to affect the transcription of Nrf2 and Nrf2-dependent genes in PBL in vivo, which is indicative for systemic bioactivity. The consumer-relevant bilberry juice showed a different activity profile than a bilberry pomace extract [6], underlining the importance of the polyphenol source and composition. Furthermore, the DNA integrity was affected by the anthocyanin-rich beverages. Taken together, the applied red fruit juice blend (beverage 1) showed the most promising properties for further studies on long-term effects of a consumer-relevant anthocyanin-rich juice, as recently shown by Bakuradze et al. [25].

## Materials and methods

### Materials

Three different juices (beverage 1–3) were provided for testing. Red fruit juice (beverage 1, 100% fruit content) was produced from red grape, blueberries, strawberries, cranberries, chokeberries, apple, and acerola provided by Eckes-Granini GmbH (Nieder-Olm, Germany). The beverage enriched with grape skin extract (beverage 2, 20 g/L) was prepared from red grapes (*Vitis vinifera L*.) originating from France (grape varieties Cabernet Sauvignon, Merlot, Syrah, and Grenache) provided by Wild GmbH & Co. KG (Heidelberg, Germany). Bilberry juice (beverage 3, 100% fruit content) was prepared from bilberries (*Vaccinium myrtillus L*.) originating from Eastern Europe and provided by Ernteband GmbH (Winnenden, Germany). The two bilberry drinks consumed in the study were prepared from two different concentrations of the above mentioned bilberry juice (beverage 3a = 100 mL juice with 600 mL water-sucrose solution; beverage 3b = 300 mL juice with 400 mL water-sucrose solution).

The total anthocyanin contents of the beverages were analyzed chromatographically using HPLC–UV/VIS-system as previously described by Mueller et al. [26]. Quantification was carried out in triplicate (as cyanidin-3-O-glucoside equivalents) using peak areas detected at 540 nm and based on external calibration using the reference substance malvidin-3-glucoside. Values of ° Brix for each beverage were determined with an *Abbé* refractometer (Carl Zeiss, Jena, Germany). The analysis of total phenolics (measured by *Folin*–*Ciocalteu* method) as well as citric acid in the beverages were performed by the beverage producers with standard lab methods.

### Ethical approval

This study was approved by the local ethic committee of Rhineland-Palatine, Mainz, Germany (no 837.013.14 (9252-F)) and conducted as open trial. Written consent forms were obtained from all volunteers prior to their inclusion in the study.

### Study design/volunteers

**Fig. 4:**
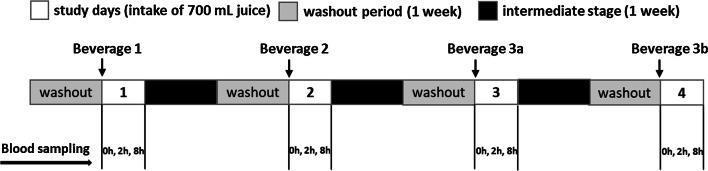
Design of the human pilot intervention study conducted as open trial. Beverage 1: red fruit juice; beverage 2: beverage with grape skin extract; beverage 3a: 100 mL bilberry juice + 600 mL water-sucrose solution; beverage 3b: 300 ml bilberry juice + 400 mL water-sucrose solution

Male, healthy non-smoking volunteers were recruited at the University of Kaiserslautern. Exclusion criteria included smoking, body mass index (BMI) > 25, use of medication, participation at other studies, deviation of the nutritional requirements, practice of competitive sport and chronic disease. All participants were asked to maintain their usual dietary habits for the duration of the study, except for the intake of food rich in polyphenols like coffee, chocolate, red wine, tea, etc. All volunteers were informed of the objectives of the study and consent was received for their participation. Before the study started, volunteers underwent a medical examination including blood pressure measurement and analysis of standard clinical blood markers to ensure that they fulfilled the status of health required. All volunteers were healthy without any chronic diseases. Five male volunteers (BMI 22.9 ± 1.9; age 23.6 ± 1.1) who fulfilled all-inclusion criteria participated in the study. After a seven-day polyphenol-reduced diet (no consumption of fruit and vegetables, fruit juice, red wine, beer, tea, chocolate and vitamin supplements), the volunteers consumed on an empty stomach a bolus (700 mL) of the respective beverage (Fig. 4). Blood samples were collected immediately prior (0 h), and 2 h and 8 h after intake of the respective beverage at each study day. During the study days the volunteers consumed a light meal (bread roll with cheese) and a light dinner (tortellini carbonara), respectively. Only during the study with beverage from grape skin extract one of five volunteers dropped out due to incompatibility (vomiting) with the beverage. The other volunteers reported a feeling of nausea during the first hour after intake of bolus of 700 mL beverage with grape skin extract.

### Isolation of human peripheral blood lymphocytes (PBL)

7 mL of freshly collected human blood anticoagulated with EDTA was layered on 7 mL of Histopaque 1077 (Sigma Aldrich). After centrifugation for 25 min (400×*g*, without break, 25 °C), the lymphocytes were collected from the layer between the plasma and Histopaque 1077 phases and were transferred into 10 mL of RPMI 1640 medium (Gibco, Life Technologies, Carlsbad, USA) supplemented with 10% fetal calf serum (FCS) and 1% penicillin/streptomycin. Thereafter, the cell suspension was centrifuged for 10 min (250 x *g*), and the pellet was resolved in 6 mL of 10% FCS medium. Lymphocytes were repeatedly washed and centrifuged. Cells were transferred into 1 mL of RNAlater stabilization reagent (Qiagen, Hilden, Germany) and stored at -80 °C.

### RNA extraction and real-time qPCR (RT-qPCR)

Total RNA from lymphocytes was isolated using the Qiagen RNeasy Mini Kit column extraction kit (Qiagen, Hilden, Germany). RNA was quantified on a NanoDrop ND-2000 system (Thermo Fisher Scientific, Wilmington DE, USA) and stored at − 80 °C until further analysis. Complementary DNA (cDNA) was obtained by reverse transcription (QuantiTect Reverse Transcription Kit, Qiagen, Hilden, Germany) according to the manufacturer’s instruction. DNA obtained from reverse transcription was subjected to RT-qPCR using QuantiTect SYBRGreen PCR master mix (Qiagen, Hilden, Germany). The QuantiTect primer assays used were for NQO-1: Hs_NQO1_1_SG, QT00050281; HO-1: Hs_HMOX1_1_SG, QT00092645; Nrf2: Hs_NFE2L2_1_SG, QT00027384; ACTB: Hs_ACTB_1_SG, QT00095431 and GAPDH: Hs_GAPDH_2_SG; QT01192646 (Qiagen, Hilden, Germany). Primer concentrations used were according to the manufacturer’s guidelines in the QuantiTect SYBR Green PCR Handbook 11/2005 (Qiagen). PCR reaction parameters were as follows: incubation at 95 °C for 15 min and thereafter 40 cycles of denaturation at 94 °C for 15 s, annealing at 55 °C for 30 s, and extension at 72 °C for 30 s. Data of all assays were analyzed by the comparative ΔΔCT- method as the amplification efficiencies of the target and reference genes have been equal as controlled for all investigated genes. This relative quantification compares the CT-value of the target transcript to the mean CT-value of the endogenous control genes β-actin (ACTB) and GAPDH. The mean fold change in expression of the target gene was calculated using 2^−ΔΔCT^ with

$$\Delta \Delta {\text{CT}}\, = \,\left( {\Delta {\text{CT}}_{{ - {\text{target}}}} \, - \,\Delta {\text{CT}}_{{ - {\text{mean }}\beta - {\text{actin}},{\text{ GAPDH}}}} } \right).$$

### Comet assay

DNA strand breaks in whole blood were determined by the alkaline single-cell gel electrophoresis (comet assay) according to Collins et al. [27–29], with slight modifications as described previously by Bakuradze et al. [30]. The aliquots of whole blood (6 µL) were mixed with low-melting agarose applied onto a pre-coated microscope slide and submitted to the lysis (24 h, 4 °C). Thereafter, slides were washed three times in enzyme buffer, drained and covered with 50 µL of either enzyme buffer or formamidopyrimidine-DNA glycosylase (fpg-enzyme, obtained from Dr. A.R. Collins, Institute for Nutrition Research, University of Oslo, Norway) to differentiate between basic and total DNA strand breaks (basic strand breaks plus oxidized bases). After DNA unwinding (pH 13.5, 20 min, 4 °C) and horizontal gel electrophoresis (20 min, 25 V, 300 mA, 0.9 V/cm), slides were washed, stained with ethidium bromide, and analyzed using a fluorescence microscope (Zeiss Axio Imager A1, filter set 15, Carl Zeiss AG, Goettingen, Germany) and computerized image analysis (Comet Assay IV, Perceptive Instruments, Suffolk, GB), scoring 2 × 50 cells per slide. DNA migration was calculated as mean tail intensity (TI %: DNA in the comet tail in percent of total DNA).

### Statistics

Results of DNA strand breaks are reported as means and SD. The *Anderson*–*Darling* test was used for the analysis of normal distribution. Differences of parameters between the study phases (normally distributed results) were analyzed with one-sided paired *t*-test. Differences without normal distribution were analyzed by one-sided paired *Wilcoxon Signed Rank* test.

The data of RT-qPCR performed in triplicate are presented as BOX-diagrams. Values are the mean ± SD normalized to the mean of β-actin and GAPDH transcript levels and are presented as relative transcription compared to transcript levels at time point 0 h, which is set to 1 (baseline). Significant differences to baseline were calculated using the Kruskal–Wallis ANOVA (# p < 0.05) and differences between time points were also calculated using the Kruskal–Wallis ANOVA (*p < 0.05).

## Data Availability

All materials used in the present study are mentioned in section “Materials and Methods” and the data will be available upon request.

## Abbreviations

ARE: Antioxidant responsive element
BMI: Body mass index
cDNA: Complementary deoxyribonucleic acid
CT-value: Cycle threshold value
DNA: Deoxyribonucleic acid
EDTA: Ethylenediaminetetraacetic acid
et al.: *et alii*; and others
etc.: *et cetera*; and other similar things
FCS: fetal calf serum
fpg: Formamidopyrimidine-DNA-glycosylase
GAPDH: Glyceraldehyde 3-phosphate dehydrogenase
HO-1: Heme oxygenase
HPLC: High-performance liquid chromatography
Keap1: Actin-coupled Kelch like ECH-associated protein 1
mRNA: Messenger ribonucleic acid
NAD(P)H: Nicotinamide adenine dinucleotide phosphate
NQO-1: NAD(P)H quione oxidoreductase
Nrf2: Nuclear factor erythroid 2-related factor 2
PBL: Peripheral blood lymphocytes
qPCR: Quantitative polymerase chain reaction
ROS: Reactive oxygen species
RPMI: Roswell Park Memorial Institute growth medium
RT-qPCR: Real time quantitative polymerase chain reaction
SD: Standard deviation
sMaf: Small-Maf protein
TI: Tail intensity
UV/VIS: Ultraviolet-visible spectroscopy
